# Tm^3+^ Modified Optical Temperature Behavior of Transparent Er^3+^-Doped Hexagonal NaGdF_4_ Glass Ceramics

**DOI:** 10.1186/s11671-017-2167-9

**Published:** 2017-06-12

**Authors:** Chengqi E, Yanyan Bu, Lan Meng, Xiaohong Yan

**Affiliations:** 1 College of Electronic Science and Engineering, Nanjing University of Posts and Telecommunications, Nanjing, 210023 China; 2 Key Laboratory of Radio Frequency and Micro-Nano Electronics of Jiangsu Province Nanjing, Jiangsu, 210023 China; 3 College of Science, Nanjing University of Aeronautics and Astronautics, Nanjing, 210023 China; 4 School of Material Science and Engineering, Jiangsu University, Zhenjiang, 212013 China

**Keywords:** NaGdF_4_ glass ceramic, Er^3+^, Tm^3+^, Excitation power, Sensitivity

## Abstract

Er^3+^-doped and Er^3+^-Tm^3+^-co-doped transparent hexagonal NaGdF_4_ glass ceramics are fabricated via melt-quenching method. The emissions of Er^3+^-doped NaGdF_4_ glass ceramics are adjusted from the green to red by varying the concentration of Tm^3+^ ion under the excitation of 980 nm. The spectrum, thermal quenching ratio, fluorescence intensity ratios, and optical temperature sensitivity of the transparent glass ceramics are observed to be dependent on the pump power. The maximum value of relative sensitivity reaches 0.001 K^−1^ at 334 K in Er^3+^-doped NaGdF_4_, which shifts toward the lower temperature range by co-doping with Tm^3+^ ions, and has a maximum value of 0.00081 K^−1^ at 292 K. This work presents a method to improve the optical temperature behavior of Er^3+^-doped NaGdF_4_ glass ceramics. Moreover, the relative sensitivity S_R_ is proved to be dependent on the pump power of 980-nm lasers in Er^3+^-doped NaGdF_4_ and Er^3+^-Tm^3+^-co-doped NaGdF_4_.

## Background

The conversion of infrared radiation to visible light has generated much of the attention in up-conversion (UC) processes, particularly in trivalent lanthanide ions (Ln^3+^)-doped UC materials [1–5], due to wide applications in the visible detection of infrared radiation, solar cells, and optical temperature sensing [6–10]. Among these applications, optical temperature sensors based on the fluorescence intensity ratio (FIR) technique were reported as a good method to measure temperatures in nanoscales [11, 12]. Er^3+^ has been proved as excellent ions in the field of optical temperature sensors, since it has the two couples of adjacent thermally coupled energy levels (^2^H_11/2_, ^4^S_3/2_) and (^2^D_7/2_, ^4^G_9/2_), whose relative emission intensities are strongly dependent on the temperature [13]. Santos et.al investigated the maximum sensitivity of optical temperature sensing using up-conversion fluorescence emissions was 0.0052/°C in Er^3+^-Yb^3+^ co-doped Ga_2_S_3_:La_2_O_3_ chalcogenide glass [14]. León-Luis et.al researched that the temperature sensor had highest sensitivity of 0.0054 K^−1^ based on the Er^3+^ green up-converted emission in a fluorotellurite glass [15]. Du et al. disclosed that the Er^3+^/Yb^3+^-co-doped Na_0.5_Gd_0.5_MoO_4_ nanoparticles had a maximum sensitivity of 0.00856 K^−1^ which is independent on the dopant concentration [16]. Zheng et al. observed five-photon up-conversion emissions of Er^3+^ for optical temperature sensing which had highest sensitivity was 0.0052 K^−1^ [17]. However, those articles were reported the sensitivity of Er^3+^-doped optical temperature material which are mainly affected by host matrix and lacked the research of influence on excitation power. In fact, the intensity of the thermally coupled energy level will vary with the intensity of the excitation power. Wang et al. found that the thermal quenching ratio and temperature sensitivity from thermally coupled energy levels of Er^3+^-doped transparent Sr_0.69_La_0.31_F_2.31_ glass ceramics were dependent on the pump power [18]. Bednarkiewicz’s group observed that the highest sensitivity value was dependent on the pump power for LiYbP_4_O_12_:0.1%Er^3+^ nanocrystals [19]. Similar result has been reported in Er^3+^-doped Y_2_SiO_5_ powders [20]. The optical thermometry at different excitation power was different, since the fluorescence intensity ratios were affected by the excitation powers. Thus, it is necessary to explore the optical temperature behavior at the different excitation powers.

Among the reported host materials, NaGdF_4_ nanocrystals have been confirmed as an excellent luminescent host matrix for various optically active Ln^3+^ in optical temperature sensor due to their relative low phonon energy and excellent chemical stability [21, 22]. Based on the couple thermally coupled energy levels ^2^H_11/2_ and ^4^S_3/2_ of Er^3+^ ion, the optical temperature properties of Er^3+^-doped NaGdF_4_ was reported [23]. However, the abovementioned work did not consider influence of excitation power to the optical temperature property of Er^3+^-doped NaGdF_4_. The optical temperature property of the Er^3+^ ions depends on the relative changes in the green emission intensity of thermally coupled energy levels ^2^H_11/2_ and ^4^S_3/2_ level. The luminescent of Er^3+^ ions was adjusted by Tm^3+^ ions through the energy transfer from Er^3+^ ions to Tm^3+^ ions [24–28]. Thus, the optical property of Er^3+^-doped NaGdF_4_ glass ceramics may be adjusted by the introduction of the Tm^3+^ ions.

In this paper, Er^3+^ single-doped and Er^3+^-Tm^3+^-co-doped hexagonal NaGdF_4_ glass ceramics were fabricated to illustrate the abovementioned issues. It is found that the luminescent of Er^3+^-doped NaGdF_4_ glass ceramics is tuned from green to red by controlling the concentration of Tm^3+^ ions. The effects of doping Tm^3+^ ions on thermal quenching ratio, population mechanism of thermally coupled levels, and temperature sensitivity are also observed by using the different excitation powers. It was observed that the optical temperature sensitivity of Er^3+^-doped and Er^3+^-Tm^3+^-co-doped NaGdF_4_ glass ceramics remained substantially increase with the increase of excitation power to the lower temperature field and reached the maximum sensitivity under 322.4 mW/cm^2^ excitation.

## Methods

The glass ceramics samples with mole composition of 70.1SiO_2_-4.3Al_2_O_3_-1.8AlF_3_-2.3Na_2_CO_3_-18.5NaF-(2.4-x)Gd_2_O_3_-0.6Er_2_O_3_-xTm_2_O_3_ (*x* = 0, 0.05, 0.1, 0.15, 0.2) were prepared by melt-quenching method, which were labeled as the NGF1, NGF2, NGF3, NGF4, and NGF5, respectively. High pure reagents of SiO_2_, Al_2_O_3_, AlF_3_, Na_2_CO_3_, NaF, Gd_2_O_3_, Er_2_O_3_, and Tm_2_O_3_ were used as raw materials. Accurately weighed 20 g batches of raw materials were ground in a mortar with fully mixed and then melted in a covered corundum crucible at 1600 °C for 45 min. The melts were cast quickly into a brass mold plates and pressed it. The obtained glass ceramics were annealed at 700 °C for 20 h to form transparent ceramics through a crystallization process in the annealing furnace. All samples were polished optically for further characterization. For a better comparison of the role of Tm^3+^ ions, the NGF1 and NGF3 are used for mainly contrast sample.

Structures of the samples were investigated by X-ray diffraction (XRD) using XTRA (Switzerland ARL) equipment provided with Cu tube with Kα radiation at 1.54056 nm. The shape and size of the samples were observed by a transmission electron microscope (JEOL JEM-2100). Luminescence spectra were obtained by an Acton SpectraPro SP-2300 Spectrophotometer with a photomultiplier tube equipped with the xenon lamp as the excitation sources. Different temperature spectra were obtained using an INSTEC HCS302 Hot and Cold System.

## Results and Discussion

**Fig. 1:**
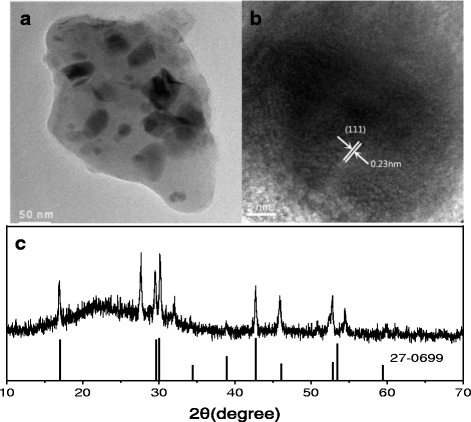
(**a**) TEM and (**b**) HRTEM micrograph images of NGF3. **c** XRD pattern of the NGF3 (JCPDS 27-0699)

The structural properties of Er^3+^-Tm^3+^-co-doped transparent NaGdF_4_ glass ceramics are studied by the transmission electron microscope (TEM), the high-resolution transmission electron microscope (HRTEM) images, and XRD, as shown in Fig. 1. It could be found that the dark spherical or irregular block nanocrystals were lying on the gray background and the size of NaGdF_4_ crystallite is about 30–55 nm, as shown in Fig. 1a. In Fig. 1b, the HRTEM image shows lattice fringes with an observed interplanar distance is about 0.23 nm, it can be attributed to the (111) crystal plane of NaGdF_4_ crystals. As shown in Fig. 1c, the position and intensity of all diffraction peaks could be readily assigned as hexagonal phase NaGdF_4_ based on the standard XRD pattern (JCPDS 27-0667), which indicates that the hexagonal phase NaGdF_4_ with a crystalline nature can be readily prepared by melt-quenching method.

**Fig. 2:**
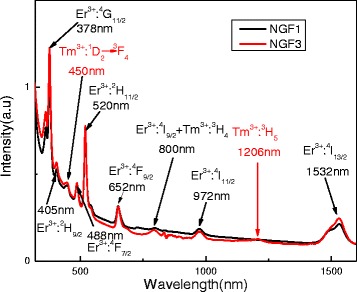
The absorption spectra of NGF1 and NGF3

The absorption spectra of NGF1 and NGF3 from 320 to 1600 nm are shown in Fig. 2. It corresponds to the transition from the ground state (except for 450 nm absorption) to the high energy level are marked in the figure. The absorption peaks of 378, 405, 488, 520, 652, 972, and 1532 nm are assigned to the transitions of Er^3+^ ions from ground state ^4^I_15/2_ to the excited state ^4^G_11/2_, ^2^H_9/2_, ^4^F_7/2_, ^2^H_11/2_, ^4^F_9/2_, ^4^I_11/2_, and ^4^I_13/2_, respectively. The absorption peak of Tm^3+^ ions have 450 and 1206 nm, which corresponds of energy transfer is ^1^D_2_→^3^F_4_ and ^3^H_5_→^3^H_6_. It is noteworthy that the shape change of peak at 800 nm absorbs wavelengths after doping Tm^3+^ ions; it may be absorbed by Er^3+^ ions and Tm^3+^ ions together. The absorption around 800 nm in the co-doped samples may be originating from the transitions Er^3+^:^4^I_15/2_→^4^I_9/2_ and Tm^3+^:^3^H_6_→^3^H_4_, respectively.

**Fig. 3:**
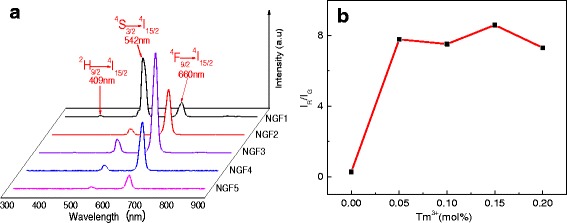
(**a**) The luminescence spectra and (**b**) red to green intensity ratio of 1%Er3+,x%Tm3+-co-doped NaGdF4 (*x* = 0, 0.05, 0.1, 0.15, 0.2)

The room temperature up-converted luminescence spectra of samples NGF1, NGF2, NGF3, NGF4, and NGF5 are investigated under the excitation of a 980-nm laser diode. The characteristic emissions of Er^3+^ ions ranging from 300 to 900 nm can be clearly observed in Fig. 3a. Emission bands located at 509 nm (NGF1), 542 nm (green, NGF3), and 660 nm (red, NGF3) are assigned to ^2^H_9/2_→^4^I_15/2_, ^4^S_3/2_→^4^I_15/2_, and ^4^F_9/2_→^4^I_15/2_ transitions of Er^3+^, respectively. As shown in Fig. 3a, with the addition of Tm^3+^ ions and the concentration increases, the 509 nm emission disappear, the 542 nm wavelength intensity decreases first and then the change is not obvious; at meanwhile, the 660 nm wavelength increases first and then decreases. In order to clearly show the relative changes between 542 nm wavelength and 600 nm wavelength intensity, the red to green intensity ratio shows in Fig. 3b. The red to green intensity ratio is increased first and then maintain a certain range of ups and downs with the Tm^3+^ ions concentration increased. In combination with Fig. 3a, b, the luminescence intensity of different wavelength has changed with the Tm^3+^ ions doping, while the position of the peak is unchanged. Therefore, Tm^3+^ ions have the effect of modified luminescence in Er^3+^-doped NaGdF_4_ glass ceramics.

**Fig. 4:**
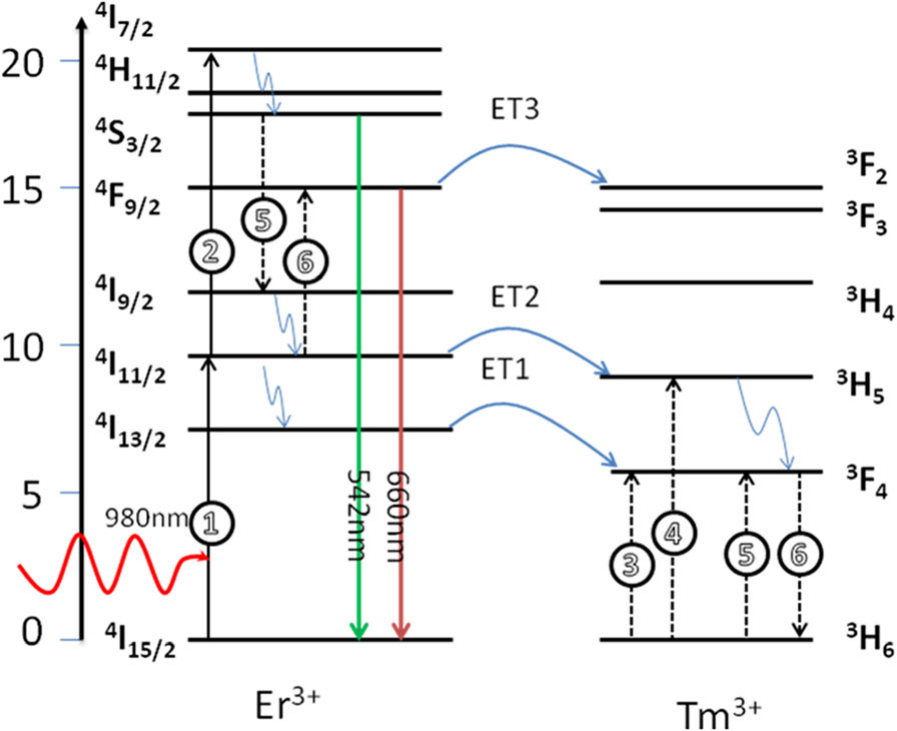
The energy level diagram showing the UC mechanism in NGF3

In order to analyzing the Tm^3+^ modified luminescence, the energy level diagram and the photoluminescence mechanism are illustrated in Fig. 4. In Er^3+^ single-doped NaGdF_4_, the 509 nm, 542 nm (green), and 660 nm (red) emission bands are observed through the transitions from ^2^H_9/2_, ^4^S_3/2_ and ^4^F_9/2_ states to ^4^I_15/2_ state, respectively. By co-doping Er^3+^ and Tm^3+^ ions in NaGdF_4_, under the 980 nm excitation, the absorption of 980 nm photons results in direct excitation of Er^3+^ ions from the ground ^4^I_15/2_ state to the excited station ^4^I_11/2_ state through a ground-state absorption (GSA) process. Then, Er^3+^ ions in the ^4^I_11/2_ state are promoted to the higher station ^4^F_7/2_ state through an excited-state absorption (ESA). After a series of nonradioactive relaxation (NR) from ^4^I_7/2_, the 542 nm (green), 660 nm (red) emission bands are observed through the transitions from ^4^S_3/2_ and ^4^F_9/2_ states to ^4^I_15/2_ state, respectively. And the green emission is reduced by an energy transfer (ET) from Er^3+^ to Tm^3+^ (5, Fig. 4): Er^3+^ (^4^S_3/2_)+Tm^3+^ (^3^H_6_)→Er^3+^ (^4^I_9/2_)+Tm^3+^ (^3^F_4_) [29]. In contrast, the population of ^4^F_9/2_ level is based on the ET processes as follows (6, Fig. 4): Er^3+^ (^4^I_11/2_)+Tm^3+^ (^3^F_4_)→Er^3+^ (^4^F_9/2_)+Tm^3+^ (^3^H_6_), which had already been confirmed [25, 30]. There are two important energy levels of 660 nm emission enhancement, Er^3+^ (^4^I_11/2_) and Tm^3+^ (^3^F_4_); the population of Er^3+^ (^4^I_11/2_) is through NR process from Er^3+^ (^4^I_9/2_); however, we found that Tm^3+^ (^3^F_4_) populated may be via three kinds of ET: the first (ET1, Fig. 4) is Er^3+^ (^4^I_13/2_)→Tm^3+^ (^3^F_4_); the second (ET2, Fig. 4) is Er^3+^ (I_11/2_)→Tm^3+^ (^3^H_5_) with subsequent NR from ^3^H_5_ (Tm^3+^) to ^3^F_4_ (Tm^3+^); and the third is previously mentioned energy transfer of green emission depopulation: Er^3+^ (^4^S_3/2_)+Tm^3+^ (^3^H_6_)→Er^3+^ (^4^I_9/2_)+Tm^3+^ (^3^F_4_). Combined with Figs. 3a and 4, the green emission drastically reduced with the Tm^3+^ ions doped; the ET of Er^3+^ (^4^S_3/2_)+Tm^3+^ (^3^H_6_)→Er^3+^ (^4^I_9/2_)+Tm^3+^ (^3^F_4_) may dominate the population of Tm^3+^ (^3^F_4_). And the red emission is quenched at the large Tm^3+^ concentration. It can be ascribed to the ET(ET3, Fig. 4): ^4^F_9/2_ (Er^3+^)→^3^F_2_(Tm^3+^).^30^ Combined with the above analysis, we can divide the energy transfer of Er^3+^-Tm^3+^ luminescence systems into two parts: (a) the excited state ^4^I_11/2_ state from the ground-state absorption and then through an excited-state absorption to the higher station ^4^F_7/2_ state by Er^3+^, through finally nonradiative relaxation from ^4^I_7/2_, the 542 nm (green), 660 nm (red) emission bands are observed; (b) the population of red-emitting and the depopulation of green-emitting can be attributed to an energy loop, Er^3+^ (^4^S_3/2_) →Er^3+^ (^4^I_9/2_) →Er^3+^ (^4^I_11/2_) →Tm^3+^ (^3^F_4_) →Er^3+^ (^4^F_9/2_), which implements the modified luminescence of Tm^3+^ ions.

**Fig. 5:**
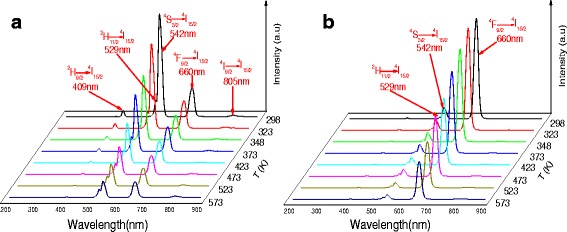
UC emission spectra of (**a**) NGF1 and (**b**) NGF3 in the wavelength range of 200–900 nm at various temperatures

The temperature sensing properties based on the luminescence emissions at 509, 529, 542, 660, and 805 nm of Er^3+^ single-doped (NGF1) and the luminescence emissions at 529, 542, and 660 nm of Er^3+^-Tm^3+^-co-doped NaGdF_4_ glass ceramics (NGF3) have been shown in Fig. 5, with the temperature ranging from 298 to 573 K, respectively. The two green up-conversion emissions bands at about 529 and 542 nm correspond to the ^2^H_11/2_→^4^I_15/2_ and ^4^S_3/2_→^4^I_15/2_ transitions of Er^3+^, respectively. The 509, 660, and 805 nm emissions correspond to the ^2^H_9/2_→^4^I_15/2_, ^4^F_9/2_→^4^I_15/2_ and ^4^I_9/2_→^4^I_15/2_ transitions of Er^3+^, respectively. With the increased of the temperature, it can be found that the emission intensities of ^4^S_3/2_ level decrease markedly. The ^2^H_11/2_ level may be also populated from the ^4^S_3/2_ level by thermal excitation, due to the thermal population and depopulation at high temperature [31]. The relative population of the “thermally coupled” ^2^H_11/2_ and ^4^S_3/2_ levels follows a Boltzmann-type population distribution, which has already been confirmed [32, 33], leading to variation in the transitions of ^2^H_11/2_→^4^I_15/2_ and ^4^S_3/2_→^4^I_15/2_ of Er^3+^ at the elevated temperature.

The thermal quenching ratio (*R*_Q_) is a key parameter to evaluate the affection of temperature on luminescence quenching [16]. The *R*_Q_ of emission band with temperature change is defined as follow:

1 $$ {R}_Q=1-\frac{I_T}{I_0} $$

**Fig. 6:**
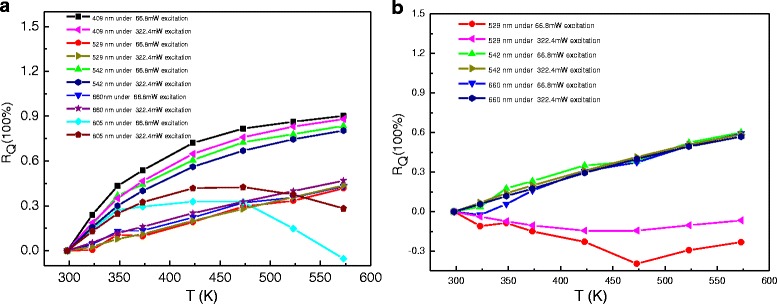
Thermal quenching ratios (*R*Q) of (**a**) NGF1, (**b**) NGF3 at low 66.8 mW/cm^2^ excitation power and at high 322.4 mW/cm^2^ excitation power

Here, *I*_*T*_ is luminescence intensity at different temperature *T*, and *I*_0_ is luminescence intensity at room temperature. The values of *R*_*Q*_ for the 409, 529, 542, 660, and 805 nm emissions of NGF1 and NGF3 show in Fig. 6 with 66.8 and 322.4 mW/cm^2^ excitation power. In Fig. 6a, with the temperature increase, the value of *R*_*Q*_ in 529 nm grows slowly than the value in 542 nm, which means emission intensity of 529 nm reduce slowly than emission intensity of 529 nm. In Fig. 6b, it shows a different trend with the increase of temperature. The value of *R*_*Q*_ at 542 nm emission band increases with temperature increase. Oppositely, the value of *R*_*Q*_ of the 529 nm emission band shows some negative values and decreases firstly and then increases with increasing temperature, which means that the ^2^H_11/2_ state is populated thermally at high temperature [34]. In Fig. 6a, the values of *R*_*Q*_ for the 409 nm emissions increase with temperature increase quickly. Compared with Fig. 6a, b at 660 nm, we could obverse that with the addition of Tm^3+^ ions, *R*_*Q*_ become a relatively large positive value, which means Er^3+^-Tm^3+^-co-doped NaGdF_4_ at 660 nm luminescence with temperature was changed significantly. The intensity of 800 nm emissions can be enhanced a lot by the increase of temperature and the decrease of excitation power in Fig. 6a, but it does not appear in Er^3+^-Tm^3+^-co-doped NaGdF_4_.

**Fig. 7:**
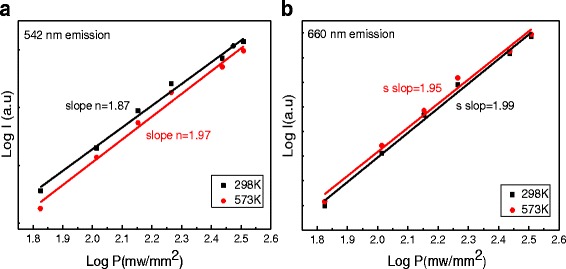
Log–log plots of intensity and pumping power for (**a**) 542 nm, (**b**) 660 nm emissions at 298 and 573 K in NGF3

To explore the origin of green emission and red emission of Er^3+^ ions at high temperatures, the relation between UC emission intensity *I* and laser light intensity *P* is expressed as:

2 $$ I\propto {P}^n $$

where *I* is the emission intensity, *P* is incident pump power, and *n* is the number of pump photons absorbed in the up-conversion process [35]. Figure 7 shows log-log plots of up-conversion intensity and pumping power for green and red at the different temperatures in NGF3. The slopes of fitted lines for 542 and 660 nm emissions change little at two temperature points of 298 and 573 K, and all values of *n* are less than 2 but greater than 1, indicating that 524 and 660 nm emissions come from two-photon up-conversion process regardless of the high temperature or low temperature.

**Fig. 8:**
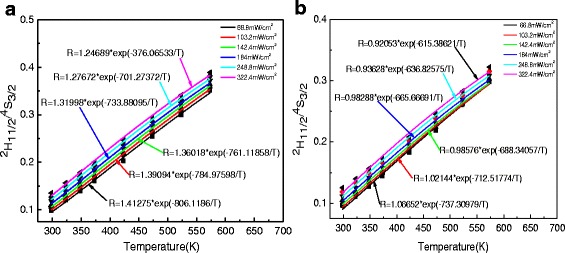
Excitation power-dependent emission intensity ratio glass ceramics of 2H11/2/4S3/2 on (**a**) NGF1 and (**b**) NGF3

In summary, two adjacent energy levels, the upper ^2^H_11/2_ level and the lower ^4^S_3/2_, can relatively change with temperature increase, which is fitting the Boltzmann distributing law, and it may be used to be as thermally coupled levels [36]. According to the theory in [16] and [23], the population ratio of ^2^H_11/2_ to ^4^S_3/2_ from thermally coupled levels of Er^3+^ is defined as:

3 $$ R=\frac{I_{\mathrm{U}}}{I_{\mathrm{L}}}= A{\mathrm{e}}^{\frac{-\varDelta E}{K_{\mathrm{B}} T}} $$

where *A* is a fitting constant that depends on the experimental system and intrinsic spectroscopic parameters; △*E* is the fitting energy difference between thermally coupled levels; *K*_B_ is the Boltzmann constant; *T* is the absolute temperature. The luminescence intensity ratio between *I*_U_ and *I*_L_ will change regularly with the temperature increase. A function relation between the luminescence intensity ratio and temperature can be determined through fitting some data points at different temperatures. The temperature-dependent fluorescence intensity ratios between the ^2^H_11/2_ and ^4^S_3/2_ of Er^3+^ in NGF1 and NGF3 samples from 298 to 573 K are shown in Fig. 8 under different excitation power. The experimental data are fitted by Eq. (3). It can be observed that the fittings agree well with the experimental data. The curve value of *R* is dependent on excitation power whether NGF1 or NGF3. It means that the fluorescence intensity ratios of the coupled levels of ^2^H_11/2_ and ^4^S_3/2_ susceptible to the pumping power in Er^3+^ single-doped and Er^3+^-Tm^3+^-co-doped NaGdF_4_ glass ceramics. Comparing Fig. 8b with Fig. 8a, under the same excitation power, it can be seen that the curve matching formula is not the same, suggesting that the population ratio of ^2^H_11/2_ to ^4^S_3/2_ was changed after doped Tm^3+^ ions.

**Fig. 9:**
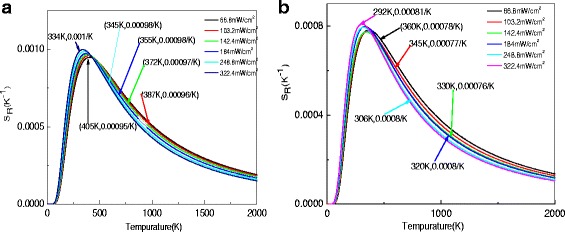
Excitation power-dependent relative sensitivity *S*R of (**a**) NGF1 and (**b**) NGF3

It is important to investigate the sensing sensitivity for further understand the temperature response of NGF1 and NGF3. The sensitivity of optical thermometry is the rate of change of *R* in response to the variation of temperature [37, 38]. The relative sensitivity *S*_R_ and the absolute sensitivity *S*_A_ are defined as:

4 $$ {S}_R=\frac{dR}{dT}= R\frac{\varDelta E}{K_{\mathrm{B}}{T}^2} $$

5 $$ {S}_A=\frac{1}{R}\frac{dR}{dT}=\frac{\varDelta E}{K_{\mathrm{B}}{T}^2} $$

where the △*E* is the energy difference between thermally coupled levels, *K*_B_ is the Boltzmann constant, *T* is the absolute temperature, and *R* is the luminescence ratio between the two thermally coupled levels [39]. Figure 9 depicts the curves of *S*_R_ of NGF1 and NGF3 samples dependent on temperature under different excitation power. Two samples show the high sensitivity at low excitation. The maximum *S*_R_ value of Er^3+^-doped NaGdF_4_ is estimated to be 0.001 K^−1^ at 334 K, while Er^3+^-Tm^3+^-co-doped NaGdF_4_ has the maximum *S*_R_ value that is 0.00081 K^−1^ at 292 K. Moreover, it is worth noting that the sensitivity peak shifts toward the lower temperature range after doping with Tm^3+^ ions.

From Fig. 9, the slopes of fitted lines for NGF1 and NGF3 are increased first and then slowly decrease with the increase of the temperature range from 0 to 2000 K, revealing that NGF1 and NGF3 can monitor a wide range of temperature. It can be clearly seen that with the addition of Tm^3+^ ions, the maximum sensitivity and the maximum sensitivity temperature are changed. Compared to NGF1 which has maximum sensitivity in temperature is about 334 K, NGF3 has maximum sensitivity in the lower temperature than in NGF1 that is about 292 K. It means Tm^3+^ ions can change the sensitivity and temperature measurement range. And it is very sensitive to measure temperature from 334 to 405 K by using the fluorescence intensity ratio of the NGF1 under excitation power from 322.4 to 66.8 mW/cm^2^. This means that Er^3+^-doped NaGdF_4_ can be used for intermediate temperature measurements. As can be seen from Fig. 9b, NGF3 has a high sensitivity at a low temperature of about 292 K. It is well known that most of the up-conversion rare earth ion-doped optical temperature materials exhibit superior sensitivity at moderate to high temperatures [40–42]. There are very few reports of optical thermometry around room temperature. Thus, NGF3 is suitable for monitoring the temperature around 20 °C. One can find that the values of *S*_R_ decrease with increase excitation powers basically in NGF1, but it first decrease and then increase with increase excitation powers in NGF3. The largest *S*_R_ appears when the excitation power is 322.4 mW/cm^2^. In addition, it can be observed that the temperature of the location about the maximum sensitivity is close to the lower temperature range as the excitation power increases. Thus, a general rule can be obtained in NGF1 and NGF3, which are more sensitive for temperature measurement in lower temperature environments as the excitation power increases. The NGF1 not only has maximum of *S*_R_ larger than NGF3 but also has the value of *S*_R_ that is more and corresponds to ordinary rules with the increase of excitation power than NGF3. Thus, the Er^3+^-doped NaGdF_4_ is a better candidate for optical temperature sensors than Er^3+^-Tm^3+^-co-doped NaGdF_4_ by considering the stabilities induced by temperature and excitation powers. According to Eq. (4), the sensitivity is determined by the energy difference (△*E*) between thermally coupled levels. Thus, the energy difference (△*E*) in NGF1 and NGF3 glass ceramics is greater than some other RE (rare earth ion)-doped materials, which leads to the higher sensitivity of NGF1 and NGF3 glass ceramics. In order to compare the sensitivity with various rare ions for optical thermometry, some of the reports of sensitivities of various rare earth ions are presented in Table 1. It shows that the sensitivity of Er^3+^-doped NaGdF_4_ glass ceramics is well than some other rare earth ion-doped material. So, it further explains that Er^3+^-co-doped NaGdF_4_ glass ceramic will be a good candidate for high-performance optical thermometry.

**Table 1:** Values of sensitivity for various rare earths are presented, and the involved transitions from thermally coupled levels as well as temperature range are included

Rare earth ions	Host	Transitions	Temperature range (K)	*S*_A_	Ref.
Er^3+^	NaGdF_4_	^2^H_11/2_,^4^S_3/2_→^4^I_15/2_	298–593	806/T^2^	This work
Er^3+^,Tm^3+^	NaGdF_4_	^2^H_11/2_,^4^S_3/2_→^4^I_15/2_	298–593	737/T^2^	This work
Tm^3+^,Yb^3+^	NaNbO_3_	^1^G_4_,^3^F_2,3_,^3^H_4_→^3^H_6_	293–353	93.53/T^2^	[43]
Tm^3+^,Yb^3+^	Y_2_O_3_	^1^D_2_→^3^F_4_,^3^H_4_→^3^H_6_	10–300	566.91/T^2^	[44]
Ho^3+^	In-Zn-Sr-Ba	^5^F_4_/^5^S_2_→^5^I_8_,^5^I_7_	20–300	181.64/T^2^	[45]
Ho^3+^	TeO_2_	^5^F_4_/^5^S_2_→^5^I_8_	265–440	255/T^2^	[46]

## Conclusions

In summary, Er^3+^-doped NaGdF_4_ and Er^3+^-Tm^3+^-co-doped NaGdF_4_ glass ceramics were prepared by a melt-quenching method and subsequent heating. The samples were investigated through XRD, TEM, and luminescence spectra measurement. Under laser excitation of 980 nm, these glasses strongly emitted light in the visible region, ranging from green to red. A visible emission which can be tuned from the green to the red color by varying the Tm^3+^ ion concentration is achieved under the 980 nm excitation. Meanwhile, the emission intensities of the Er^3+^-doped and Er^3+^-Tm^3+^-co-doped transparent NaGdF_4_ glass ceramics were found to be temperature dependent. It was found that the spectrum structure, thermal quenching ratio, fluorescence intensity ratio, and sensitivity from thermally coupled levels were strongly dependent on the change of pump powers. Optical temperature sensing of the Er^3+^-doped and Er^3+^-Tm^3+^-co-doped NaGdF_4_ transparent glass ceramics in the temperature that ranges from 298 to 573 K is studied. The maximum value of relative sensitivity (*S*_R_) is 0.001 K^−1^ at 334 K under 322.4 mW/mm^2^ excitation. And it shifts toward the lower temperature range and has a maximum value of 0.00081 K^−1^ at 292 K after doped with Tm^3+^ ions. The results indicate that the Er^3+^-doped and Er^3+^-Tm^3+^-co-doped NaGdF_4_ transparent glass ceramics may be good candidates for the temperature sensor.

## Abbreviations

△E: Energy difference
ESA: Excited-state absorption
ET: Energy transfer
FIR: Fluorescence intensity ratio
GSA: Ground-state absorption
HRTEM: High-resolution transmission electron microscope
Ln^3+^: Trivalent lanthanide ions
NGF1: 0.6%Er^3+^-doped NaGdF_4_ glass ceramics
NGF2: 0.6%Er^3+^-0.05%Tm^3+^ co-doped NaGdF_4_ glass ceramics
NGF3: 0.6%Er^3+^-0.1%Tm^3+^ co-doped NaGdF_4_ glass ceramics
NGF4: 0.6%Er^3+^-0.15%Tm^3+^ co-doped NaGdF_4_ glass ceramics
NGF5: 0.6%Er^3+^-0.2%Tm^3+^ co-doped NaGdF_4_ glass ceramics
NR: Nonradioactive relaxation
RE: Rare earth ion
R_Q_: Thermal quenching ratio
S_A_: Absolute sensitivity
S_R_: Relative sensitivity
TEM: Transmission electron microscope
UC: Up-conversion
XRD: X-ray diffraction
